# Effects of Particle Hydrophobicity, Surface Charge, Media pH Value and Complexation with Human Serum Albumin on Drug Release Behavior of Mitoxantrone-Loaded Pullulan Nanoparticles

**DOI:** 10.3390/nano6010002

**Published:** 2015-12-25

**Authors:** Xiaojun Tao, Shu Jin, Dehong Wu, Kai Ling, Liming Yuan, Pingfa Lin, Yongchao Xie, Xiaoping Yang

**Affiliations:** 1 Department of Pharmacy, School of Medicine, Hunan Normal University, Changsha 410013, China; xiaojtao@126.com (X.T.); lingkai520@126.com (K.L.); yuanlimingtian@126.com (L.Y.); xieychao@sohu.com (Y.X.); 2 Department of Gastroenterology, Taihe Hospital, Hubei University of Medicine, Shiyan 442000, China; jinshu76@sohu.com; 3 Department of Radiology, Taihe Hospital, Hubei University of Medicine, Shiyan 442000, China; wdhsyth@163.com; 4 Fujian Vocational College of Bioengineering, Fuzhou 350300, China; linpingfa@163.com

**Keywords:** pullulan nanoparticles, degree of substitution, HSA, surface charge

## Abstract

We prepared two types of cholesterol hydrophobically modified pullulan nanoparticles (CHP) and carboxyethyl hydrophobically modified pullulan nanoparticles (CHCP) substituted with various degrees of cholesterol, including 3.11, 6.03, 6.91 and 3.46 per polymer, and named CHP_−3.11_, CHP_−6.03_, CHP_−6.91_ and CHCP_−3.46_. Dynamic laser light scattering (DLS) showed that the pullulan nanoparticles were 80–120 nm depending on the degree of cholesterol substitution. The mean size of CHCP nanoparticles was about 160 nm, with zeta potential −19.9 mV, larger than CHP because of the carboxyethyl group. A greater degree of cholesterol substitution conferred greater nanoparticle hydrophobicity. Drug-loading efficiency depended on nanoparticle hydrophobicity, that is, nanoparticles with the greatest degree of cholesterol substitution (6.91) showed the most drug encapsulation efficiency (90.2%). The amount of drug loading increased and that of drug release decreased with enhanced nanoparticle hydrophobicity. Nanoparticle surface-negative charge disturbed the amount of drug loading and drug release, for an opposite effect relative to nanoparticle hydrophobicity. The drug release in pullulan nanoparticles was higher pH 4.0 than pH 6.8 media. However, the changed drug release amount was not larger for negative-surface nanoparticles than CHP nanoparticles in the acid release media. Drug release of pullulan nanoparticles was further slowed with human serum albumin complexation and was little affected by nanoparticle hydrophobicity and surface negative charge.

## 1. Introduction

Many drugs with highly efficient action are dysfunctional as treatment because of the fast drug metabolism and a peak-valley reaction. With fast drug metabolism, the drug reaches peak concentrations quickly, which usually leads to severe drug toxicity, and the peak-valley reaction is inefficient or invalid. To avoid these problems, a slow-release drug strategy has been developed to preserve the drug-efficient concentration in the blood by a sustained release effect and has become a major research field [1,2].

Nanoparticles have novel nano-size structure with the capacity of capturing small molecule drugs to form novel nano-drug preparations or formulations [3,4,5]. Nano-drug formulations improve slow-drug release capacity, which is vital for enhancing the drug curative effect and reducing toxic side effects [6,7,8,9].

Polymeric amphiphiles such as polysaccharides modified with hydrophobic groups can spontaneously form a nano-spherical structure in aqueous media with a hydrophobic micro-core and a hydrophilic shell [10,11,12]. A nanoparticle hydrophobic core can load a hydrophobic drug via a hydrophobic interaction [13,14]. The hydrophobic group of nano-materials affects the amount of drug loading and the drug release efficiency [15]. It plays a major role in the size of the formed nanoparticles, related to the final drug function [16]. The carboxyl group has been conjugated to polymeric amphiphiles to obtain self-aggregated nanoparticles with negative surface charge in aqueous solution to confer unique stability and liver targeting ability as compared with counterparts [17,18]. Adding the carboxyl group can affect the self-assembly process of formed nanoparticles by affecting the sizes formed and the drug-loading amount [17]. Therefore, nano-drug release with a nanoparticle negative-surface charge should be studied for *in vivo* drug efficiency function.

Besides the nanoparticle hydrophobicity and surface charge, the pH value in the medium has a critical role in nano-drug release [18,19]. The drug release amount is greater with pH < 7 than ≥ 7 [20]. This finding is vital for cancer treatment because nano-drug release is better with an acidic environment [21,22].

Human serum albumin (HSA) is a major soluble globular protein in the circulatory system and is often coated on the surfaces of many nanoparticles with nano-drug *in vivo* treatment [23,24]. It affects nano-drug release and also the targeted tissue, thereby leading to altered nano-drug efficiency [25,26]. Therefore, nano-carriers with precise properties should be designed according to certain biologic environments for the desired slow-drug release function.

**Figure 1:**
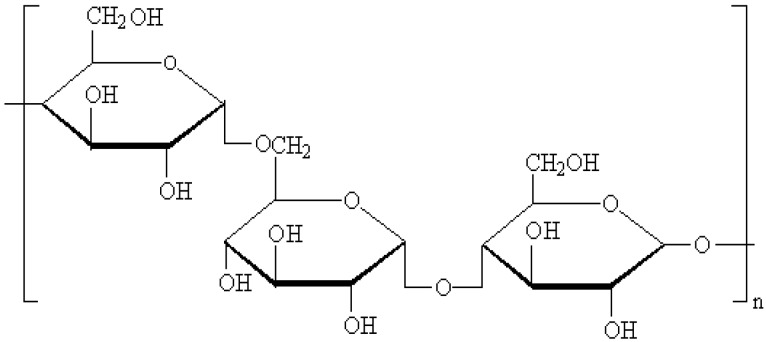
Chemical structure of pullulan.

**Figure 2:**
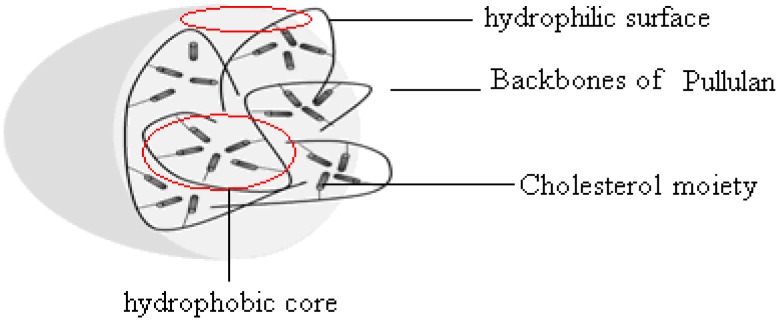
Structural graph of pullulan nanoparticle.

Pullulan is a linear polysaccharide with maltotriose repeating unites jointed by α-1, 6-linkages (Figure 1), that is safe, non-toxic, hydrophilic, and biodegradable [27,28]. Its many hydroxyl groups can be modified with hydrophobic groups such as a long-chain alkyl group and cholesterol to form monodispersed self-aggregated pullulan nanoparticles [29,30,31]. Pullulan nanoparticles include a hydrophobic core with cholesterol moiety and a hydrophilic surface with pullulan chain (Figure 2). Pullulan nanoparticles can be used as a small-molecule carriers, or macromolecule carriers such as protein and gene carriers, widely studied for medical applications [32,33,34]. However, besides their superior targeting function and drug efficiency, pullulan nanoparticles have not been studied for their effect on nano-drug slow release.

Therefore, we investigated the effect of nanoparticle properties, release-media pH value, and HSA complexation on drug-release behaviors of these nano-drug carriers in a biological application. Especially, the drug release of pullulan nanoparticles with HSA complexation is important to explore because these data will be helpful for further *in vivo* application.

**Figure 3:**
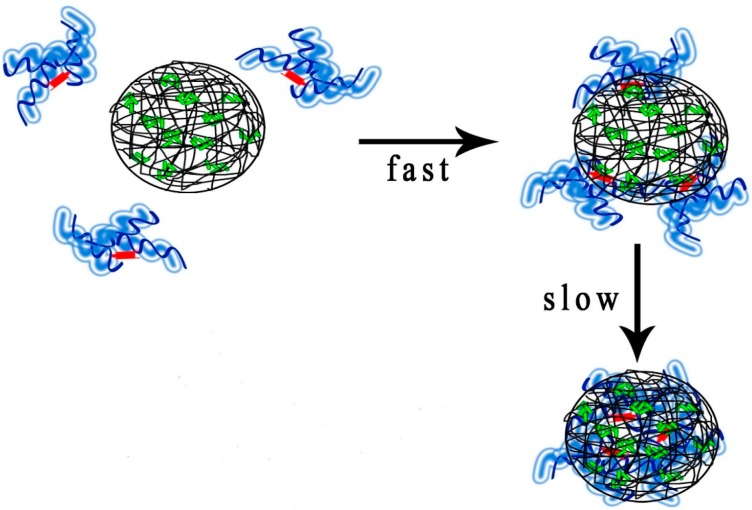
Sketch illustration of human serum albumin (HSA) complexation with pullulan nanoparticles.

We grafted cholesterol to hydroxyl groups of pullulan by esterification and changed the degree of cholesterol substitution to obtain different types of hydrophobically modified pullulan (CHP). To design a negative-surface charge nanoparticle, carboxyethyl pullulan hydrophobically-modified with cholesterol (CHCP) was synthesized via an esterification reaction. CHP and CHCP conjugates can self-aggregate nanoparticles with different hydrophobicity and negative surface charge by a dialysis method. The prepared pullulan nanoparticles were characterized to analyze particle composition including surface charge, size and shape, and illustrate the effect of cholesterol group and carboxyethyl groups on drug release. We measured drug encapsulation, loading capacity and sizes of pullulan nanoparticles with different properties and observed the morphology via transmission electron microscopy (TEM). The effect of nanoparticle hydrophobicity and surface charge on the drug loading amount and drug release in phosphate buffered saline (PBS) was studied to clarify the application as a drug carrier. We collected cumulative drug release rate (%) of pullulan nanoparticles with different properties and charge surface in weak acid and strong release media and studied alterations in drug release amount with acid intensity by nanoparticle properties. We also studied HSA complexation with CHP nanoparticles. This complexation can be divided into two processes: HSA fast-surface coating and slow inter-insertion (Figure 3) [35]. For HSA complexation with nano-carrier processes, drug release from the loaded pullulan nanoparticles was further sustained [36]. We studied the nano-drug release of pullulan nanoparticles with different properties on HSA complexation and discuss whether HSA complexation is vital for nano-drug release. Finally, we demonstrate a drug delivery paradigm with CHP and mitoxantrone as a model drug. Our nano-drug slow preparation can be used for further *in vivo* efficacy study.

## 2. Results and Discussion

### 2.1. Properties of Pullulan Nanoparticles

**Figure 4:**
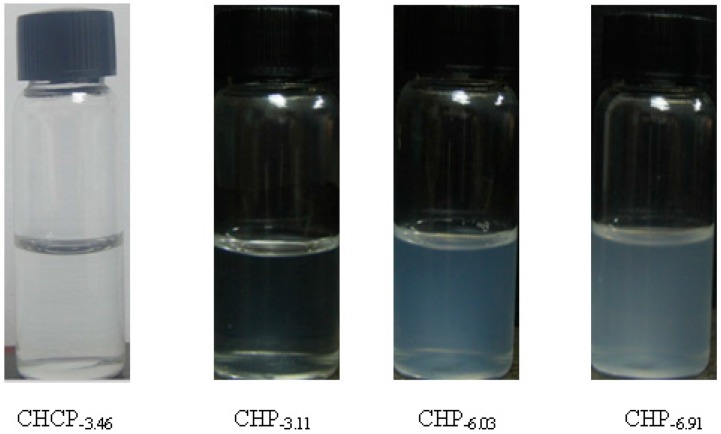
The optical appearance of pullulan nanoparticles with different hydrophobicity and surface charge.

**Figure 5:**
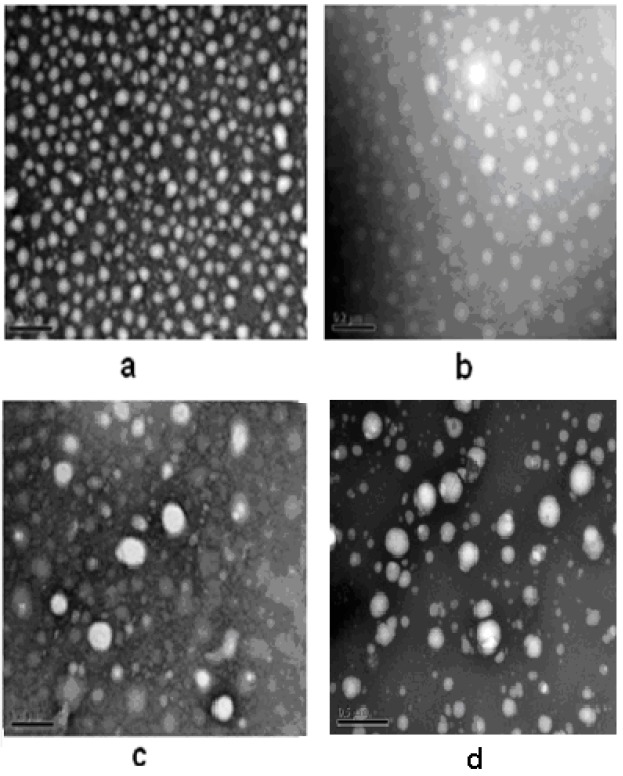
Transmission electron microscopy (TEM) of CHP_−6.91_ (**a**); CHP_−6.03_ (**b**); CHP_−3.11_ (**c**) and CHCP (**d**) nanoparticles.

**Figure 6:**
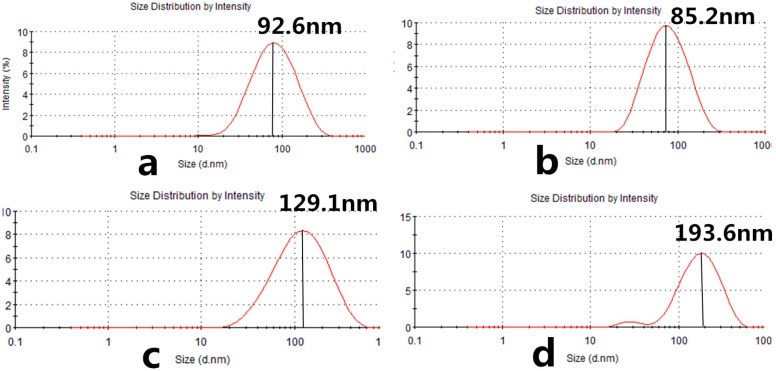
Size distribution of CHP_−6.91_ (**a**); CHP_−6.03_ (**b**); CHP_−3.11_ (**c**) and CHCP (**d**) nanoparticles.

**Figure 7:**
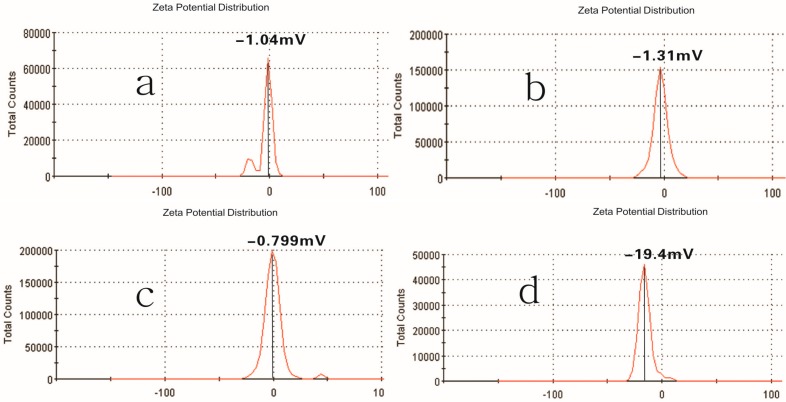
Zeta potential of CHP_−6.91_ (**a**); CHP_−6.03_ (**b**); CHP_−3.11_ (**c**) and CHCP (**d**) nanoparticles.

Pullulan self-aggregated nanoparticles with three kinds of hydrophobic properties were prepared and collected at the volume (10 mL) of the concentration 8.28 × 10^−6^ mol/L. Figure 4 shows the optical appearance of pullulan nanoparticles and Figure 5 shows that pullulan nanoparticles were regularly spherical in shape. The milky light of CHP_−6.91_ particles with a high cholesterol substitution was stronger than that of CHP_−6.03_, CHP_−3.11_, and CHCP nanoparticles by the order of light reduction. The optical appearance of the pullulan nanoparticles depended degree of on cholesterol group substitution: the more substitution lead to more milky-like. We measured nanoparticle size to explore a relationship with the cholesterol group degree of substitution. Figure 6 showed that peak size was 92.62, 85.20 and 129.1 nm for CHP_−3.11_, CHP_−6.03_ and CHP_−6.91_, respectively. However, the average size was successively 83.11, 101.9 nm and 110 nm, and decreased with the increasing of degree of cholesterol group substitution. CHP nanoparticles carried a fractional charge (about −2–0 mV) for ease in adhering to interparticles, which enlarged the average particle size. Figure 7 compares zeta potential of CHP_−3.11_ and CHCP nanoparticles. The degree of cholesterol group substitution was similar, but the surface charge was greater (−19.9 mV). The mean size was larger for CHCP than CHP_−3.11_ nanoparticles (160 *vs.* 110 nm). In fact, the difference would be larger if we considered the effect of adhesion in interparticles on size. Therefore, the cholesterol group was vital for the formed nanoparticle size, which was affected by nanoparticle surface charge. Hydrophobic interaction among hydrophobic-dependant groups results in the formation of compact hydrophobic cores that can load a hydrophobic drug into a nanoparticle core [29,37,38]. We found that pullulan conjugates with different hydrophobic and carboxyethyl groups affected nanoparticle size because the nanoparticle size affected nano-drug tissue distribution and final drug efficiency. Nanoparticle hydrophobicity and charge amount depending on hydrophobic and carboxyl groups, also affected the protein coating, which is related to drug release [39,40,41].

### 2.2. Drug-Loading of Pullulan Nanoparticles

**Figure 8:**
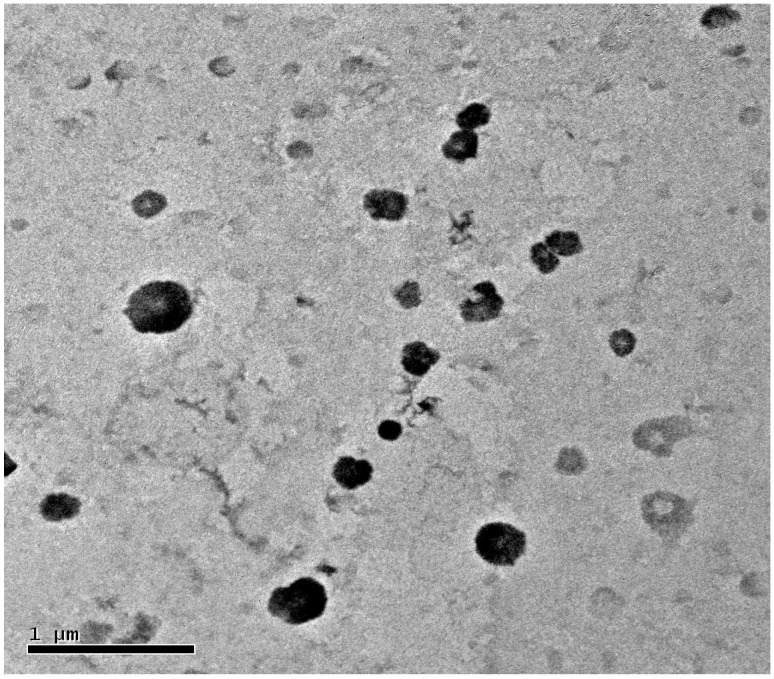
TEM of CHP_−6.03_ nanoparticles loading mitoxantrone.

CHP can self-aggregate to form nanoparticles in aqueous media with a hydrophobic micro-core because of the hydrophobic interactions among modified cholesterol groups, and hydrophilic sugar chain as an external shell [35,37]. Mitoxantrone as an anticancer model drug with benzene and cyclic structure show a strong hydrophobic property. It can be adsorbed to cholesterol groups to form nanoparticle hydrophobic core in the nanoparticle self-aggregating process [35,37]. Mitoxantrone with many hydrophilic groups, such as OH and NH group, can be also adsorbed to the external sugar chain of pullulan nanoparticles by intermolecular force. Hydrophobic adsorption has a stronger force and plays a main role in drug loading, and the intermolecular force is weak, for a role in supplementary drug loading. We loaded mitoxantrone into pullulan nanoparticles by the dialysis method. 4 mg mitoxantrone, 40 mg pullulan conjugates, and 3 mL triethylamine were dissolved in 20 mL dimethyl sulfoxide with triethylamine, and the mixture was put into a dialysis bag. In the dialysis process, cholesterol groups can self-assemble to form a nanoparticle core via a hydrophobic force with the mitoxantrone adsorption. It was assumed that the more cholesterol groups of the prepared conjugates, the greater the drug loading for the formed pullulan nanoparticles. Table 1 proved our assumption, showing encapsulation efficiency from 52.4% to 90.2% and loading capacity from 4.45% to 10.1% with cholesterol substitution from 3.11 to 6.91. However, CHCP conjugates with cholesterol substitution 3.46, greater than for CHP_−3.11_, can form negative-charge nanoparticles with encapsulation efficiency and loading capacity of 50.1% and 4.25% as compared with 52.4% and 4.45% for CHP_−3.11_ nanoprticles. Thus, besides the hydrophobic force contribution to drug loading, the carboxyethyl group can improve the hydrophobic drug-loading behavior. The mean size of mitoxantrone-loaded pullulan nanoparticles was larger than their nano-carrier, from 128.5 to 162.3 nm for CHP_−3.11_ to CHP_−6.91_, respectively. The drug-loaded size of pulllan nanoparticles depended on degree of cholesterol substitution. CHCP nanoparticles had the largest mean size, 213.5 nm. TEM graph (Figure 8) demonstrated that, mitoxantrone-loaded pullulan nanoparticles had a regularly spherical shape.

**Table 1:** Characteristics of self-aggregated nanoparticles loading mitoxantrone.

Sample	C/D (*w*/*w*) ^a^	EE (%) ^b^	LC (%) ^c^	*D*_h_ (nm) ^d^
CHP_−3.11_	1/10	52.4 ± 2.03	4.45 ± 0.26	162.3 ± 4.6
CHCP	1/10	50.1 ± 1.92	4.25 ± 0.23	213.5 ± 6.2
CHP_−6.03_	1/10	85.1 ± 1.92	8.82 ± 0.23	142.6 ± 4.2
CHP_−6.91_	1/10	90.2 ± 2.43	10.1 ± 0.32	128.5 ± 3.6

Data are mean ± standard deviation (SD). ^a^ mitoxantrone/CHP or CHCP (mg/mg); ^b^ encapsulation efficiency determined by ultraviolet (UV) spectrophotometry at 608 nm; ^c^ loading capacity determined by UV spectrophotometry at 608 nm; ^d^ Size and size distribution determined by the dynamic laser light-scattering.

### 2.3. Effect of Nanoparticle Properties on Drug Release

**Figure 9:**
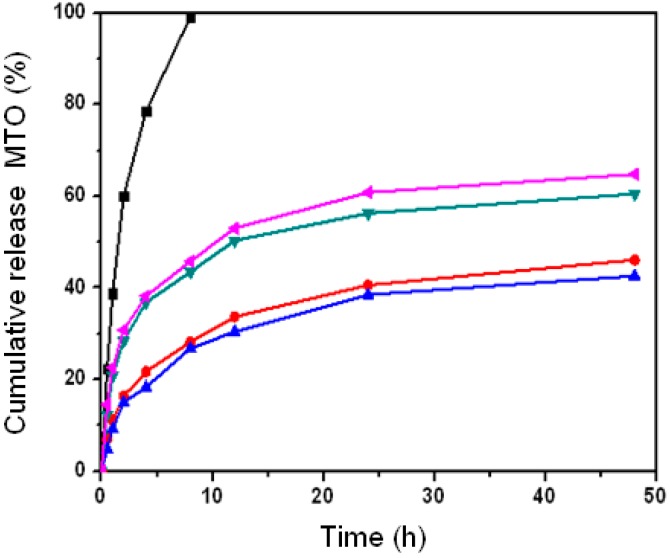
The mitoxantrone (MTO) release of pullulan nanoparticles in phosphate buffered saline (PBS) at 37 °C *in vitro* (■: free mitoxantrone, ▼: CHP_−3.11_, ◄: CHCP, ●: CHP_−6.03_, ▲: CHP_−6.91_).

Pullulan nanoparticles load mitoxantrone in two patterns: drug surface adsorption and hydrophobic core embedding, which determines whether nano-drug release is fast or slow [36]. A weak molecule adsorption causes fast drug separation and results in fast nano-drug release. However, a strong hydrophobic force and sugar-chain shell obstruction from the loading drug in the hydrophobic nanoparticle core induces a slow drug release. These two patterns are shown in the drug release graphs in Figure 9. Most free drug was released within 12 h, which indicates a fast-release pattern of the nude drug. In contrast, drug release amounts were 42.6%, 46.1%, 60.5% and 64.8% for CHP_−6.91_, CHP_−6.03_, CHP_−3.11_ and CHCP_−3.46_ nanoparticles, respectively, at 48 h. Besides drug loading amount, drug release amount was associated with nanoparticle hydrophobicity. Nanoparticles with greater cholesterol substitution (higher hydrophobicity) showed slower drug release. This rule was not observed for CHCP nanoparticles with degree of substitution 3.46. The total drug released was 64.8% for CHCP nanoparticles and 60.5% for CHP_−3.11_ nanoparticles, so the release effect was slower for CHP than CHCP nanoparticles.

### 2.4. Effect of Nanoparticle Property on Drug Release with Different pH

**Figure 10:**
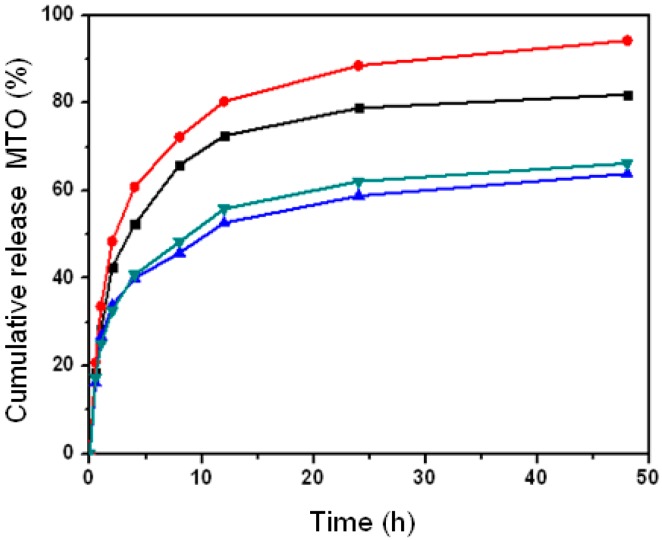
The mitoxantrone (MTO) release from pullulan nanoparticles in PBS buffer (pH 6.8) at 37 °C *in vitro* and acetate buffer (pH 4.0) (▲: CHP_−3.11_, ▼: CHCP; pH 6.8), (■: CHCP, ●: CHP_−3.11_; pH 4.0).

Since nanoparticle hydrophobicity regularly affected the drug release as described in above experiments, we chose CHCP and CHP_−3.11_ nanoparticles to examine the effect of nanoparticle surface charge on drug release in a weak or strong acid release media. Figure 10 shows the drug release of CHCP and CHP_−3.11_ nanoparticles in the release media with pH 6.8 and 4.0. In the release media with pH 6.8, the drug release amount was 63.8% and 66.2% for CHP_−3.11_ and CHCP nanoparticles, respectively, at 48 h. In the release media with pH 7.4, the drug release amount was 60.5% and 64.8%, respectively, so a weak-acid release media favored drug release. The drug release was fast and substantially increased for release media with pH 4.0: 94.2% and 81.9% for CHP_−3.11_ and CHCP nanoparticles, respectively, at 48 h. We inferred that strong acid release media (pH 4.0) may lead to degradation of the self-aggregated nanoparticles [18,22,42]. It was because that CHP or CHCP molecules were hydrolyzed at ester bond with strong acid interaction and release time prolonging, which led the spherical structure of pullulan nanoparticles to become loose until the segment degradation. As a result of this change, the generous release of the nanoparticle core loading drug happened, which was observed as a markedly increased drug release amount, showed in Figure 10. The drug release rates for CHCP nanoparticles were 64.8%, 66.2% and 81.9% in the release media with pH 7.4, 6.8 and 4.0, respectively, as compared with CHP_−3.11_ nanoparticles, with 60.5%, 63.8% and 94.2% release, respectively. The media acid value affected the drug release for CHP more than CHCP nanoparticles, which is meaningful for rationally designing nano-drug slow-release preparations for *in vivo* application.

### 2.5. The Effect of Nanoparticle Properties on Drug Release upon HSA Complexation

**Figure 11:**
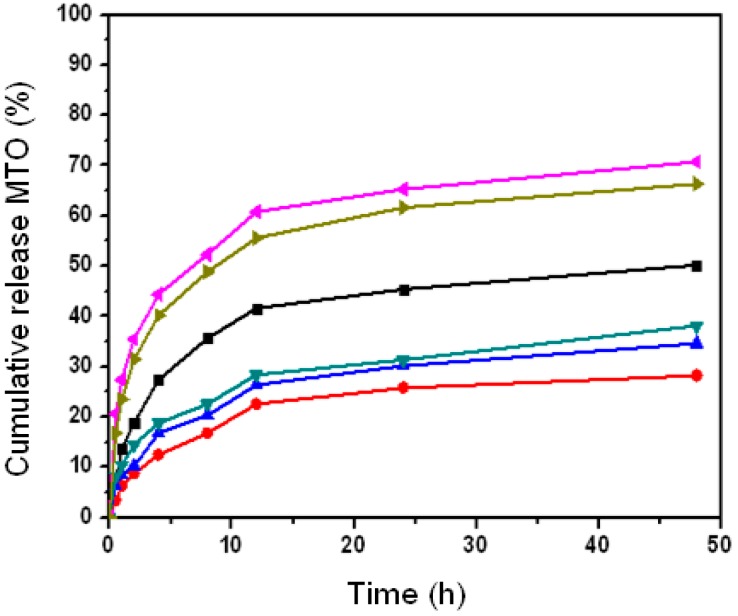
The mitoxantrone (MTO) release of pullulan nanoparticles upon human serum albumin (HSA) complexation in PBS at 37 °C *in vitro* (●: CHP_−6.91_, ▲: CHP_−3.11_, ▼: CHCP); and in the HSA release media (■: CHP_−6.91_, ►: CHP_−3.11_, ◄: CHCP).

We examined whether HSA complexation affected the drug release of nanoparticles with different properties. Figure 11 shows the drug release for CHP_−6.91_, CHP_−3.11_ and CHCP nanoparticles with HSA complexing and in the HSA release media. With HSA complexation, the drug release was 28.2%, 34.6% and 38.2% for CHP_−6.91_, CHP_−3.11_ and CHCP nanoparticles, respectively, but 42.6%, 60.5% and 64.8%, respectively, in PBS. Even for nanoparticles with a lower degree of cholesterol substitution bound to HSA molecules with weaker interaction force and less binding amount [35], the effect of HSA complexation on drug release was significant. In the HSA release media, the drug release of CHP_−6.91_, CHP_−3.11_ and CHCP nanoparticles was 50.2%, 66.4% and 70.8%, respectively, but 42.6%, 60.5% and 64.8% in PBS, so without complexation of HSA, the total amount of drug released in nanoparticles increased. Thus, HSA binding with various nanoparticle properties may be vital for designing highly efficient nano-drugs.

Use of novel nanoparticles as drug carriers has recently attracted broad attention in the drug developme nt field because of their two potential functions: targeting drug delivery and controlled slow release [43,44,45]. Nano–slow-release drug preparations have been studied widely; for example, pullulan nanoparticles with a hydrophobic core loading a hydrophobic drug showed a striking slow drug release [35,36,37]. To design a suitable slow-release nano-drug, several crucial factors including nanoparticle properties and HSA complexation must be detailed. Before the nano-drug preparation can enter the body to play a function, it cannot get eliminate all the protein coating [46,47]. Protein coating affects particle biodistribution, therapeutic efficacy, and mainly drug release amount [48,49,50]. The amount and type of protein coating is decided by nanoparticle properties [51,52]. Therefore, nanoparticle properties such as hydrophobicity and surface control the drug slow-release function. We found reduced drug release with increased nanoparticle hydrophobicity. However, drug release was enhanced with a negative-charged nanoparticle surface.

**Figure 12:**
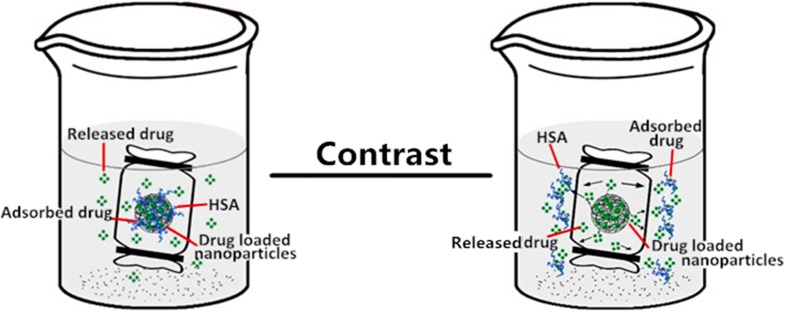
Drug released from nanoparticles upon HSA complexation and in the HSA release media.

When pullulan nanoparticles enter the body with nano-drug treatment, protein adsorption, especially HSA, may affect the slow-release function of the nano-drug preparation. HSA has high affinity for mitoxantrone [53] and can complex with pullulan nanoparticles, depending on the nanoparticle properties [35]. The drug release of pullulan nanoparticle-loaded mitoxantrone showed an intricate process with HSA fast-surface adsorption and slow nanoparticle core embedding. The slow release of pullulan nanoparticle-loaded mitoxantrone on HSA complexation was further enhanced with an intricate mechanism (Figure 12). We investigated nanoparticle hydrophobicity and surface charge to further discuss the HSA complexation effect on drug release. Even if the pullulan nanoparticle property changed the HSA binding force and amount [35,36], the drug release for all pullulan nanoparticles was slower.

In this study, the unique interaction among mitoxantrone, HSA, and pullulan nanoparticles is interesting. If HSA did not bind to pullulan nanoparticles, the drug release amount from pullulan nanoparticles could be enhanced because the fast-released drug is absorbed by HSA as shown in Figure 12. The subsequent decrease in mitoxantrone concentration near the pullulan nanoparticles favored further release. This mechanism is confirmed by the present study showing that the drug release of pullulan nanoparticles was greater in HSA than PBS media. Of course, if nanoparticles cannot bind HSA and at same time, the loaded drug in nanoparticles cannot be coated by HSA, the drug release of nanoparticles would be different. Our results have shown that novel nanoparticle properties control the *in vivo* protein absorption and drug slow-release function. Of note, we investigated only the principal extracellular protein HSA; if we consider the other factors in the human body systems interacting with nanoparticles, the drug release mechanism will more be complicated.

Besides featuring nanoparticle properties and protein binding, pullulan nanoparticles display an evidently sustained drug release depending on pH value and ion intensity of release media. Here, we discuss the drug release from pullulan nanoparticles in acid release media and analyzed the effect of nanoparticle negative charge on drug release. Drug release from pullulan nanoparticles was increased in acid media, and negative surface-charge nanoparticles showed reduced sensitivity to this action. Because the cancer cell environment is acidic and nanoparticles as a drug carrier are often loaded with anticancer drugs to treat cancer, we provide useful information for further investigation of the drug release of nanoparticles with acidic media.

To obtain a highly efficient slow-release drug carrier, the nano-drug release should be examined to design the precise treatment requirement for *in vivo* high-efficacy delivery. Further study of other factors such as ions and temperature sensitivity of materials is necessary to the *in vivo* application of nano-drug preparations.

## 3. Experimental Section

### 3.1. Materials

Cholesterol modified pullulan (CHP) were synthesized as followed [35]:

The pullulan power (0.5 g) was dissolved in 30 mL dry DMSO. The cholesterol succinate (CHS) in dried DMSO was activated by addition EDC (1.5 equiv. CHS) and DMAP (1 equiv. CHS). The mixture was reacted by stirring at 45 °C for 48 h. Then, the reactant mixture was dropped into ethanol to obtain CHP conjugate, and it was washed with ethanol, THF and diethyl ether respectively.

Cholesterol modified carboxyethyl pullulan (CHCP) were synthesized as followed:

Synthesis of carboxyethyl pullulan (CEP): Pullulan (3.0 g) was mixed with acrylic acid (1.26 mL) at molar ratio 1:1. Then, the mixture was incubated for 4 h at 50 °C with KOH solution as a catalytic agent. The reaction solution was cooled to room temperature and was placed into 500 mL ethanol. Yellowish-brown precipitation was obtained on removing the ethanol solution. It was dissolved with 40 mL distilled water, filtered, and dialyzed against 5000 mL hydrochloric acid solution (pH = 4.5 ± 0.2) for 2 days and distilled water to remove hydrochloric acid and other small substance. The CEP dialysate was collected by freezed-dried to obtain a white cotton solid.

Synthesis of carboxyethyl pullulan hydrophobically-modified with cholesterol (CHCP): NHS-activated cholesterol succinate (CSN) were synthesized according to the method which we previously described [54,55]. CEP was dissolved in 10 mL DMSO, and put into round fask in oil bath pan. CSN (CSN/glucose unit = 0.1–0.5 mmol/mmol) and (1-(3-Dimethylaminopropyl)-3-ethylcarbodiimide hydrochloride (EDC/CSN = 1.0 mmol/mmol) were dissolved in the mixture of DMSO and tetrahydrofuran. CHCP conjugate was prepared by the reaction of CSN with CEP at 45 °C according to the method as the same as CHP synthesis, shown in the above experiment.

Pullulan (20 kDa) was substituted with 3.11, 6.03, 6.91 cholesterol moieties per 100 glucose units and named CHP_−3.11_, CHP_−6.03_ and CHP_−6.91_. Pullulan (20 kDa) was substituted with 3.46 cholesterol moieties and with 10.1 carboxyethyl moieties per 100 glucose units in one CHCP molecule. HSA (fatty acid-free) was from Sigma-Aldrich (St. Louis, MO, USA). Mitoxantrone was purchased from Beijing Xinze Science and Technology Company (Beijing, China). Dialysis bags (molecular weight cut off 14,000) and other chemical reagent were obtained from Changsha Huicheng Commerce Company (Changsha, China).

### 3.2. CHP and CHCP Nanoparticle Characterization

All types of pullulan nanoparticles were prepared by the dialysis method [35,36]. Briefly, hydrophobically modified pullulan conjugates were dissolved in dimethyl sulfoxide and the mixture solution was dialyzed for 24 h to completely remove dimethyl sulfoxide. Pullulan nanoparticle solutions were passed through a membrane fliter (pore size: 0.45 µm, Millipore). The solutions were collected in bottles to obtain photos of their appearance. The size distribution and zeta potential of the pullulan nanoparticles were determined by dynamic laser light scattering at 25 °C with a BI-90US (HORIBA Ltd., Kyoto, Japan) light-scattering spectrophotometer. To observe morphology, a sample solution (0.5 mg·mL^−1^) of pullulan nanoparticles was observed by TEM with a JEM-100C (JEOL Ltd., Kyoto, Japan) electron microscope at 80 °C.

### 3.3. Mitoxantrone-Loaded Nanoparticle Characterization

Drug-loaded pullulan nanoparticles were prepared as described [31]. Briefly, 4 mg mitoxantrone and 40 mg conjugates were dissolved in 20 mL dimethyl sulfoxide, and an amount of triethylamine was added to help the drug load onto nanoparticles. Dimethyl sulfoxide and free mitoxantrone were removed by dialysis (dialysis tube, weight cutoff, 12–14 kDa, Millipore) for 9 h to obtain mitoxantrone-loaded nanoparticles. The sizes of the obtained drug-loaded CHP and CHCP nanoparticles were measured by DLS. Nanoparticle drug encapsulation efficiency and loading capacity were calculated as described [13].

### 3.4. In Vitro Drug Release of Pullulan Nanoparticles with Different Properties

The solution of mitoxantrone-loaded CHP nanoparticles (a certain amount) was put in Visking dialysis bag and dialyzed against the PBS release media at 37 °C. Then, 2 mL of the release media was collected and replaced with an equal volume of the fresh release media at pre-defined time intervals (Ti). The released amount of drug was determined by UV spectrophotometry (UV-384 plus, Molecular Devices, Thermo Fisher Scientific Inc., Waltham, MA, USA), and drug release percentage rate (*Q*%) was measured as follows [37].

(1) $$Q\%=(C_{n}\times V+V_{n}\sum_{i=0}^{i=n}C_{i})/W_{\text{drug-loading}}$$

where *C_n_* is the sample concentration at *T_n_*, *V* is the total volume of release medium, *V_i_* is the sample volume at *T_i_*, *C_i_* is the sample concentration at *T_i_* (both *V*_0_ and *C*_0_ were equal to zero), and *T_n_* is the sampling at the *N*_th_ time. The drug release amount from CHP and CHCP nanoparticles was collected to compare with the drug release feature.

### 3.5. In Vitro Drug Release of Pululllan Nanoparticles in Release Media with Different pH

Mitoxantrone release was studied *in vitro* by a dialysis method in phosphate buffered saline (PBS; pH 6.8) and acetate buffer (pH 4.0). Briefly, the solution of mitoxantrone-loaded CHCP nanoparticles was placed into dialysis tubing (dialysis tube, weight cut-off, 12–14 kDa, Millipore) and dialyzed against the release media at 37 ± 0.2 °C in an air-bath shaker at 100 rpm. Then, the drug release amount was collected to calculate the release percentage according to the above method. The drug release amount from CHP_−3.11_ nanoparticles was collected in media at different PH values to compare with the release from CHCP nanoparticles.

### 3.6. Drug Release of Pulullan Nanoparticles with HSA Complexation

CHP nanoparticles can complex with HSA and bind completely and firmly in a 9 h reaction as we previously described [35]. Therefore, we mixed an amount of HSA and drug-loaded nanoparticles to obtain the nanoparticle-drug-HSA complex, then measured the drug release as described previously. We studied the drug release of the three types of pullulan nanoparticles, CHP_−6.91_, CHP_−3.11_, CHCP, after HSA complexation. We changed the PBS release media to HSA release media at a certain concentration and analyzed the drug release of the three types of pullulan nanoparticles.

## 4. Conclusions

Nanoparticle hydrophobicity and surface charge affected the size formed, drug loading amount, and drug release of pullulan nanoparticles. The size formed and drug release of self-aggregated pullulan nanoparticles decreased with increasing cholesterol substitution. However, the drug-loading amount showed an opposite relationship. Carboxyethyl groups appeared to form a nanoparticle surface-negative charge and disturb the size formed and the drug-loading amount as well as the drug release amount, which were related to nanoparticle hydrophobicity. The amount of drug released from pullulan nanoparticles was increased in the acid media. However, the drug release was weaker from nanoparticles with a negative charge. The drug release from pullulan nanoparticles was slower with HSA complexation than in PBS and HSA media. Nanoparticle hydrophobicity and surface charge affected nano-drug release after nanoparticle-HSA complexation only slightly. Further study of nano-drug slow-release preparations with different properties is crucial to fully understand the potential application of nano-drug carriers in drug delivery.
